# Boosted Photocatalytic CO_2_ Reduction to CO with Perovskite CoTiO_3_

**DOI:** 10.3390/molecules31183281

**Published:** 2026-09-16

**Authors:** Junli Xu, Weilin Zheng, Wanpeng Sun, Zhaoyu Wang

**Affiliations:** 1 Fujian Provincial Engineering Research Center for Food Flexible Packaging Technology, Fujian Polytechnic Normal University, Fuqing 350300, China; xjl012794@163.com (J.X.); zhengweilin10@163.com (W.Z.); 2 School of Chemistry and Chemical Engineering, Henan University of Technology, No. 100 Lianhua Street, Zhengzhou High-Tech Development Zone, Henan 450001, China

**Keywords:** CoTiO_3_, photocatalytic CO_2_ reduction, CO, cocatalyst

## Abstract

In this study, we report the facile synthesis of perovskite CoTiO_3_ nanorods and their application as an efficient, noble-metal-free cocatalyst for visible-light-driven photocatalytic CO_2_ reduction to CO. Under optimized conditions (MeCN/H_2_O/TEOA, λ > 420 nm), CoTiO_3_-600 enhanced the CO yield by more than 15-fold compared to the system without CoTiO_3_, achieving 29.9 μmol of CO after 1 h of irradiation. Isotopic labeling with ^13^CO_2_ confirmed that the evolved CO originated solely from CO_2_. Mechanistic investigations revealed that CoTiO_3_ facilitates rapid electron transfer and suppresses charge recombination, as evidenced by photoluminescence quenching. Furthermore, the cocatalyst exhibited excellent stability and reusability, with no significant structural changes observed after multiple reaction cycles. Collectively, this work demonstrates that CoTiO_3_ serves as a cost-effective and robust cocatalyst, holding great promise for sustainable photocatalytic CO_2_ fixation.

## 1. Introduction

Fossil fuel depletion and the ever-increasing atmospheric CO_2_ emissions are closely linked to global climate change. These intertwined challenges have emerged as formidable issues confronting human society in recent years [1,2,3,4,5]. Consequently, extensive research efforts have been devoted worldwide to exploring clean, renewable energy sources and developing effective strategies to reduce atmospheric CO_2_ levels. Solar energy, being an inexhaustible and environmentally friendly resource, offers a compelling solution [6,7]. In particular, visible-light-driven CO_2_ conversion into energy-rich molecules has long been regarded as one of the most promising approaches to addressing the above challenges, as it not only mitigates CO_2_ emissions but also generates valuable chemicals or fuels, thereby alleviating energy shortages [8,9,10].

Despite numerous efforts devoted to the development of photocatalytic CO_2_ reduction systems, the achieved catalytic yields remain insufficient for practical applications, primarily due to the intrinsic limitations of the applied photocatalysts and cocatalysts. Cocatalysts play a crucial role in significantly enhancing the efficiency of CO_2_ photofixation reactions [11,12,13]. The cocatalysts not only suppress electron–hole recombination to enhance charge transport, but also provide extra active sites to lower both thermodynamic and kinetic barriers. Consequently, there is an urgent need to develop cost-effective, highly efficient, and stable cocatalysts for CO_2_ photoreduction that can serve as alternatives to the commonly used noble metal-based materials.

Among the numerous reported photocatalysts, perovskite oxide CoTiO_3_ has aroused much interest recently owing to its superior photoelectronic properties, visible-light-responsive band gap, and high carrier mobility. In particular, its unique ABO3 structure can increase ionic defects or oxygen vacancies via holding more cations, resulting in improved photocatalytic performances. Recently, CoTiO_3_ has been intensively investigated in the fields of energy conversion and storage [14,15,16]. Nguyen reported that the CoTiO_3/_TiO_2_ heterojunction nanocatalysts exhibit excellent photocatalytic performance for degradation of cinnamic acid, due to the formed heterojunction between CoTiO_3_ and TiO_2_ [17]. Zheng demonstrated that the constructed heterojunction in CdS@CoTiO_3_ could contribute to the photocatalytic degradation of 2,4-DCP and TC [18]. These studies imply that the CoTiO_3_ can both construct heterojunctions with other photocatalysts as well as function as cocatalysts to facilitate electron migration, thereby benefiting photocatalytic reaction.

Herein, we report a facile synthesis of CoTiO_3_ and its use as an efficient, stable cocatalyst for photocatalytic CO_2_ reduction under mild conditions. The synthesized CoTiO_3_ product was fully characterized by physical and chemical measurements. The CoTiO_3_ cocatalyst boosted the catalytic efficiency of the CO_2_-to-CO photoreduction by more than 15-fold. Isotopic labeling with ^13^CO_2_ confirmed that the produced CO originated from CO_2_. After exploring and optimizing various reaction parameters, a possible mechanism was proposed for the CoTiO_3_ promoted photocatalytic CO_2_ conversion. Furthermore, the excellent stability and reusability of CoTiO_3_ were firmly established, highlighting its great potential for constructing robust photocatalytic CO_2_ fixation systems when integrated with conventional semiconductor photocatalysts.

## 2. Results and Discussion

**Figure 1:**
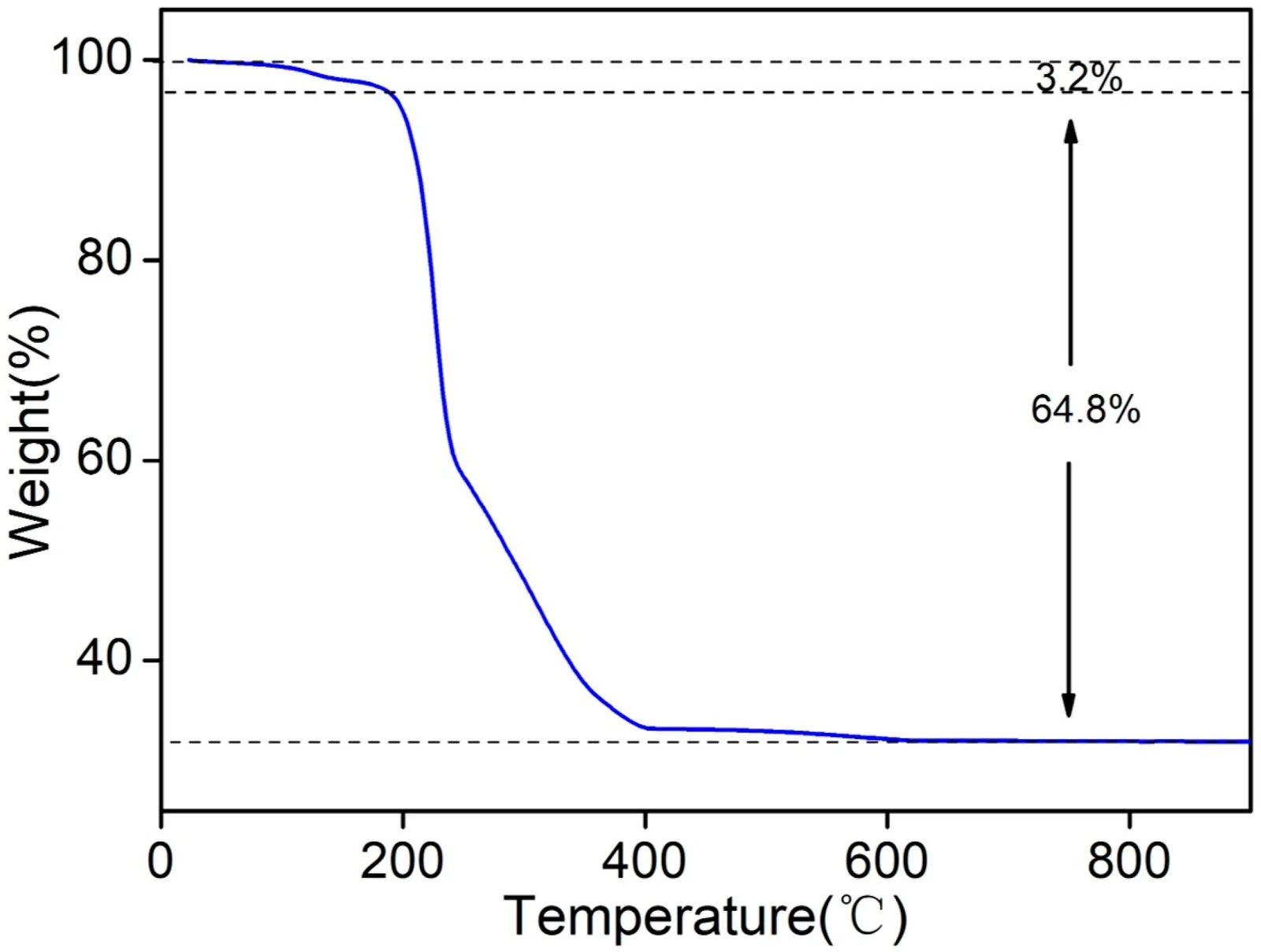
TG curve of the CoTiO_3_ nanorod precursor.

Figure 1 shows the thermogravimetric (TG) analysis curve of the CoTiO_3_ nanorods precursor. The TG curve reveals two distinct weight loss stages during the calcination process. Below 200 °C, a weight loss of 3.2% is primarily attributed to the desorption of physically adsorbed H_2_O from the surface of the cobalt titanate precursor. Another significant weight loss occurs in the range of 350–600 °C, with a weight loss rate of approximately 64.8%, which is primarily ascribed to the reaction between metal oxides to form CoTiO_3_. The TG curve of the precursor confirms that 600 °C is the minimum temperature required for the formation of CoTiO_3_ nanorods. By varying the calcination temperature, a series of samples were obtained, enabling the investigation of the effects of various calcination temperatures on the crystal structure, morphology, and other characteristics of the CoTiO_3_ samples.

**Figure 2:**
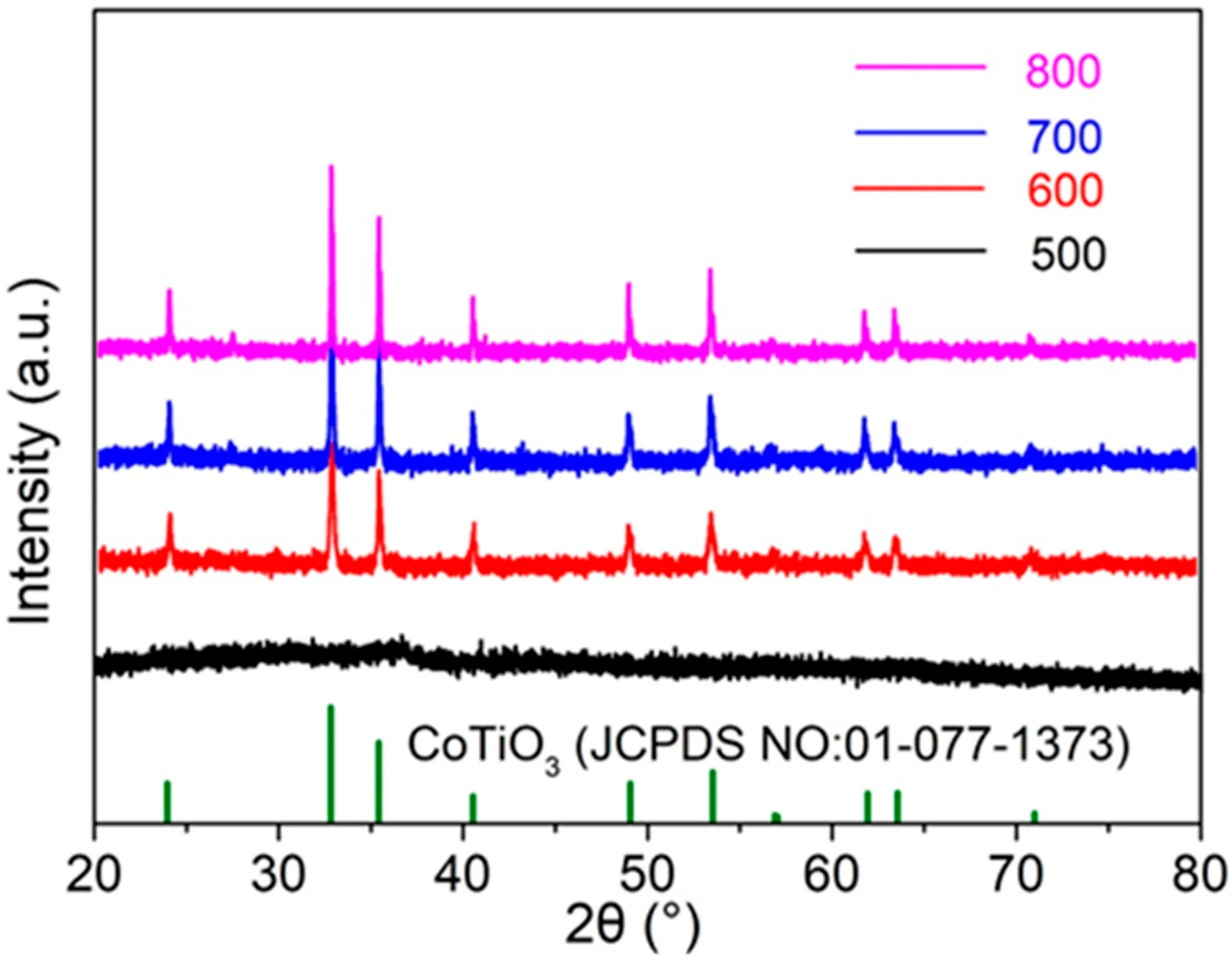
XRD patterns of CoTiO_3_ sample at different calcination temperature.

X-ray diffraction (XRD) analysis was performed to investigate the crystalline structure of CoTiO_3_ nanorods synthesized at different calcination temperatures. As shown in Figure 2, no characteristic diffraction peaks corresponding to CoTiO_3_ were observed for the sample calcined at 500 °C, indicating that the CoTiO_3_ had not yet formed and the sample remained in an intermediate state of the calcination process. This finding is consistent with the previous TG analysis results. In contrast, when the calcination temperature ranged from 600 to 800 °C, the diffraction peaks located at 23.9°, 32.8°, 35.4°, 49.1°, 53.5° and 63.5° correspond to the (012), (104), (110), (024), (116), and (300) planes of the hexagonal CoTiO_3_ (JCPDS No. 01-077-1373), respectively. No other impurity peaks were detected in the XRD patterns, indicating that pure phase CoTiO_3_ nanorods can be obtained at calcination temperatures of 600–800 °C [19]. Moreover, the diffraction peak intensities increased progressively with higher calcination temperatures, indicating that the calcination temperature plays a crucial role in determining the crystallinity of CoTiO_3_. The average crystallite sizes of the samples were about 39 nm (600 °C), 52 nm (700 °C), and 63 nm (800 °C) by using the Scherrer equation, demonstrating the high temperature result in sintering.

**Figure 3:**
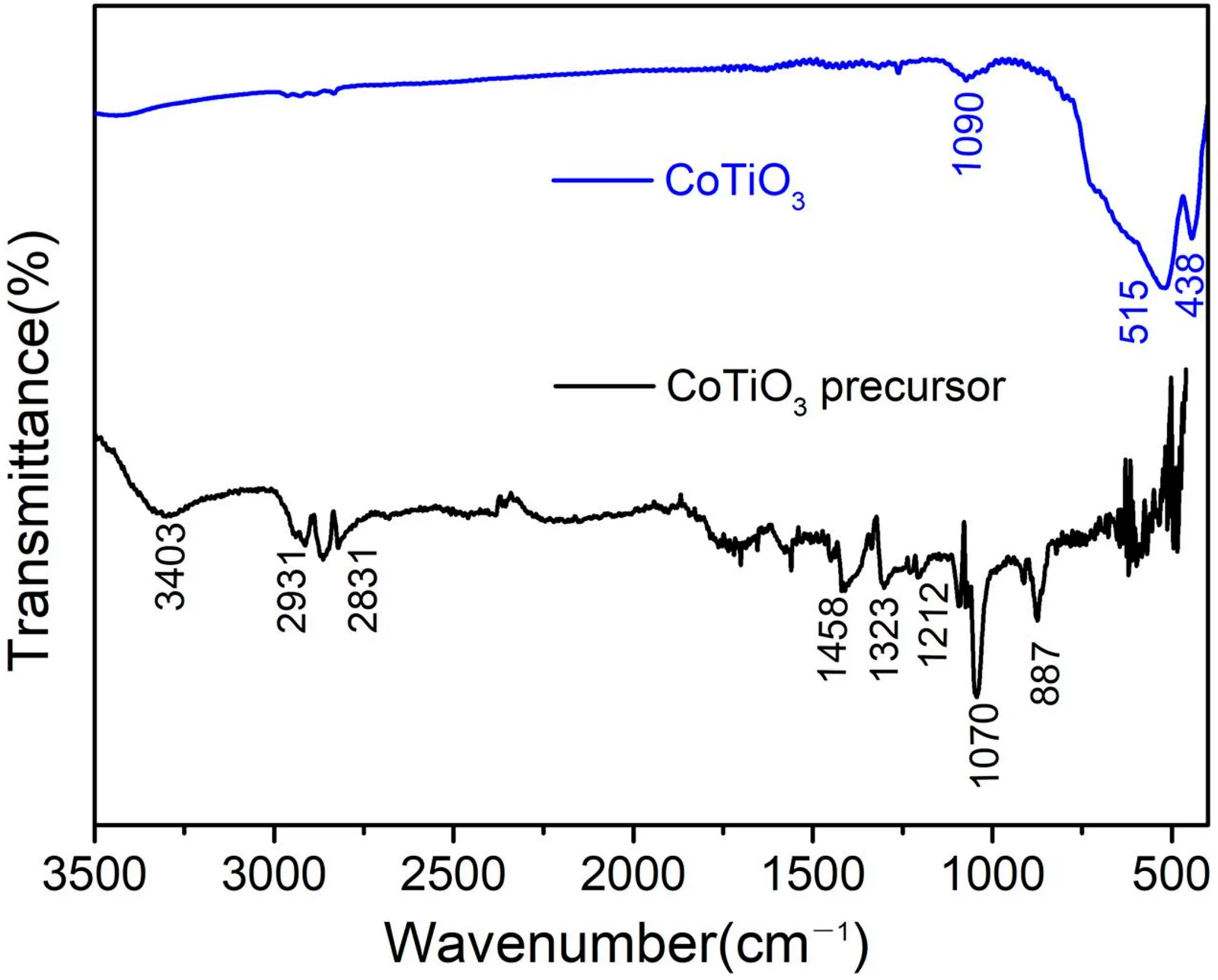
FT-IR spectra of CoTiO_3_ and precursor.

The FT-IR spectra of the CoTiO_3_ precursor are shown in Figure 3. It can be observed that the precursor exhibits strong absorption bands at 2831 and 2931 cm^−1^, which are attributed to the symmetric and asymmetric stretching vibrations of C-H bonds in -CH_2_- groups, respectively. The characteristic peaks at 3403, 1458, 1070, and 887 cm^−1^ correspond to the vibrations of C-O and O-H bonds in -CH_2_-OH groups. Meanwhile, the characteristic peaks at 1323 and 1212 cm^−1^ are assigned to the O-H and C-O absorption bands of -COOH groups. In comparison, the FT-IR spectrum of the as-synthesized CoTiO_3_ nanorods shows a weak absorption band at 1630 cm^−1^, which may arise from the O-H stretching vibration of H_2_O adsorbed on the sample surface. Additionally, the characteristic absorptions in the range of 400–800 cm^−1^ are attributed to Ti-O and Ti-O-Ti bonds, while the characteristic peak at 1090 cm^−1^ is caused by the combination of Co-O and Ti-O bonds, ultimately confirming the formation of the target CoTiO_3_ sample [17,20].

**Figure 4:**
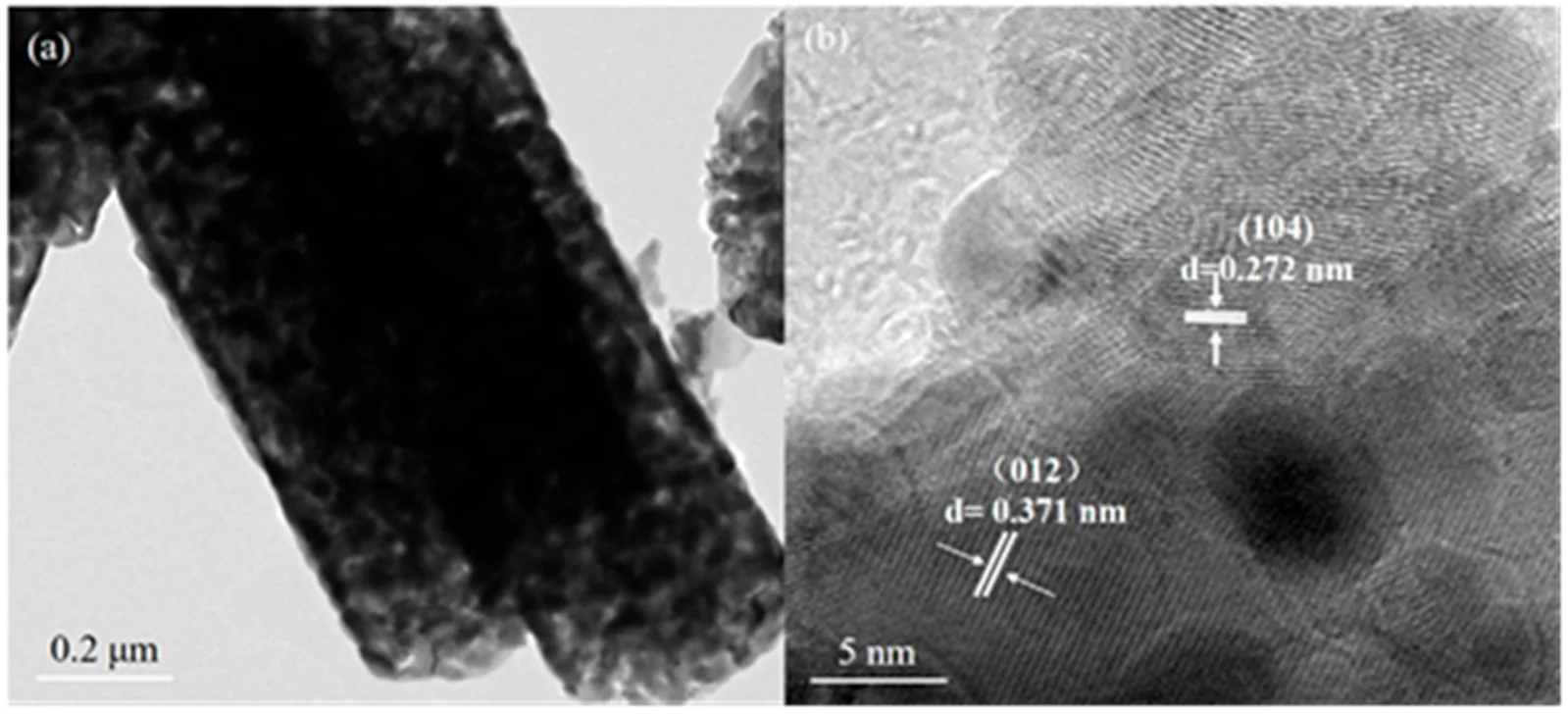
Typical TEM images (**a**) and high-resolution TEM image (**b**) of CoTiO_3_ nanorods.

Scanning electron microscopy (SEM) was employed to characterize the morphology and particle size of CoTiO_3_ samples calcined at different temperatures. For CoTiO_3_-500 (Figure S1a), the target product CoTiO_3_ had not yet formed, and the sample exhibited a relatively smooth nanorod of 700 nm in diameter and 2 μm in length. With calcination temperature increasing to 600 °C, the surface of CoTiO_3_-600 became relatively rough, exhibiting nanorods shape composed of aggregated small nanoparticles (Figure S1b). In comparison with the sample calcined at 700 °C, the size of the nanorods showed no significant variation (Figure S1c). Upon further increasing the calcination temperature to 800 °C, the nanoparticles exhibit obvious aggregation (Figure S1d). The TEM image of the CoTiO_3_-600 shows lattice fringes of 0.272 nm and 0.371 nm (Figure 4), corresponding to the (104) and (012) crystal planes of the hexagonal phase CoTiO_3_, respectively [21,22]. The CoTiO_3_-600 °C was characterized by EDX and EDX-mapping. The EDX spectrum of CoTiO_3_-600 °C revealed that the material consisted of only Co, Ti and O elements (Figure S2a), with an atomic ratio of approximately 1:1:3 (Co:Ti:O). The EDX-mapping images clearly show that Co, Ti, and O are uniformly distributed across the sample surface (Figure S2b).

**Figure 5:**
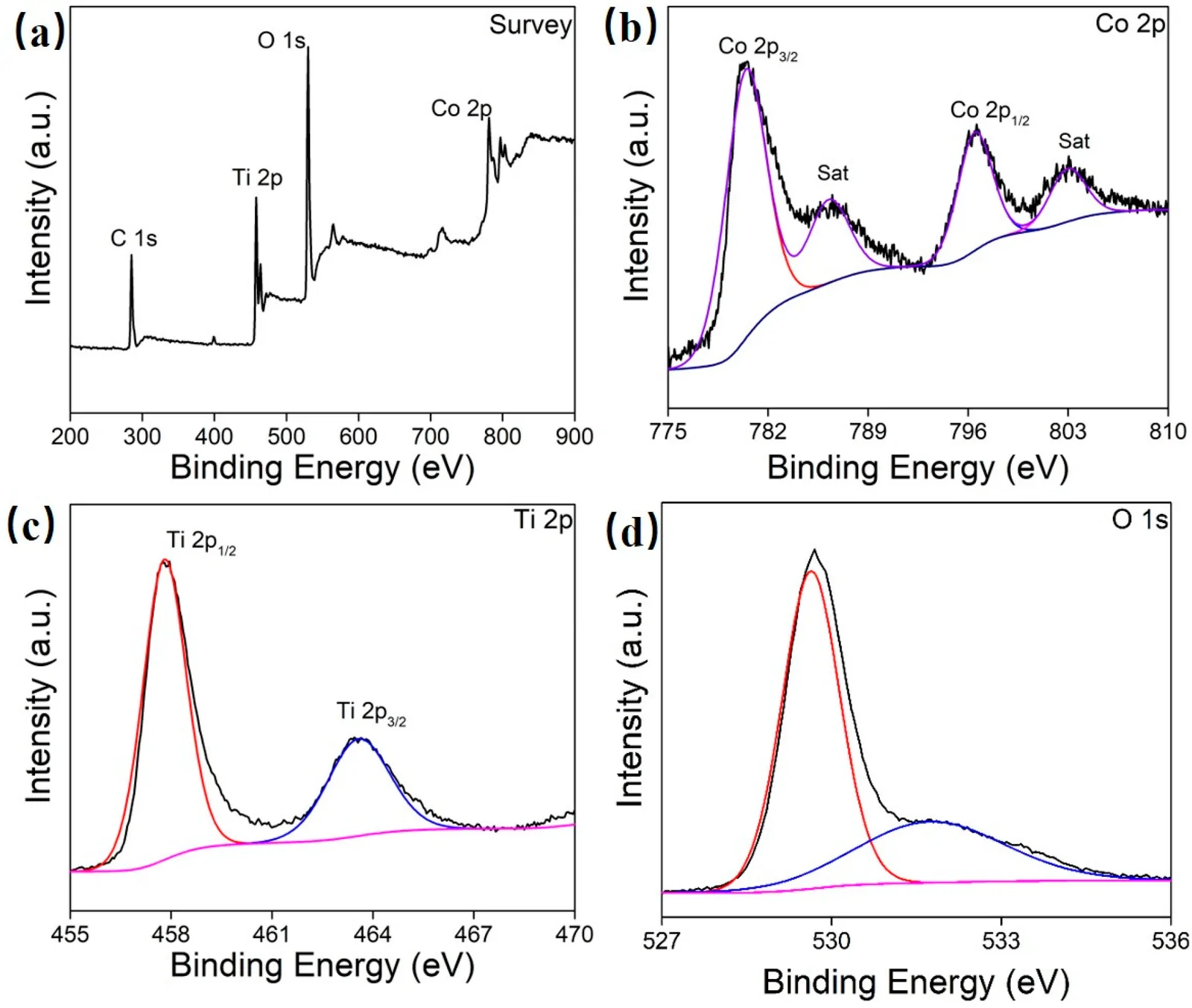
XPS spectra of CoTiO_3_ nanorods: (**a**) survey spectrum, (**b**) Co 2p, (**c**) Ti 2p, (**d**) O 1s.

XPS characterization was carried out to get insights into the oxidation state of the elements of the prepared CoTiO_3_ solid. In the survey XPS spectrum shown in Figure 5a, only signal peaks corresponding to Co, Ti, and O were detected, which is consistent with the EDX characterization results. In the high-resolution Co 2p spectrum (Figure 5b), two strong main peaks at binding energies of 780.5 eV and 796.6 eV, along with two satellite peaks, are attributed to Co 2p_1/2_ and Co 2p_3/2_, respectively [23]. In the Ti spectrum (Figure 5c), the peaks at binding energies of 457.8 eV and 463.6 eV correspond to Ti 2p_3/2_ and Ti 2p_1/2_, respectively [24]. In the O XPS spectrum (Figure 5d), the binding energies of 529.6 eV and 531.8 eV are assigned to Ti-O and Co–O bonds, as well as to O-H bonds from H_2_O adsorbed on the surface [18]. The presence of surface O-H groups is beneficial for trapping photogenerated holes, thereby generating •OH radicals, which facilitates the photocatalytic reaction.

**Figure 6:**
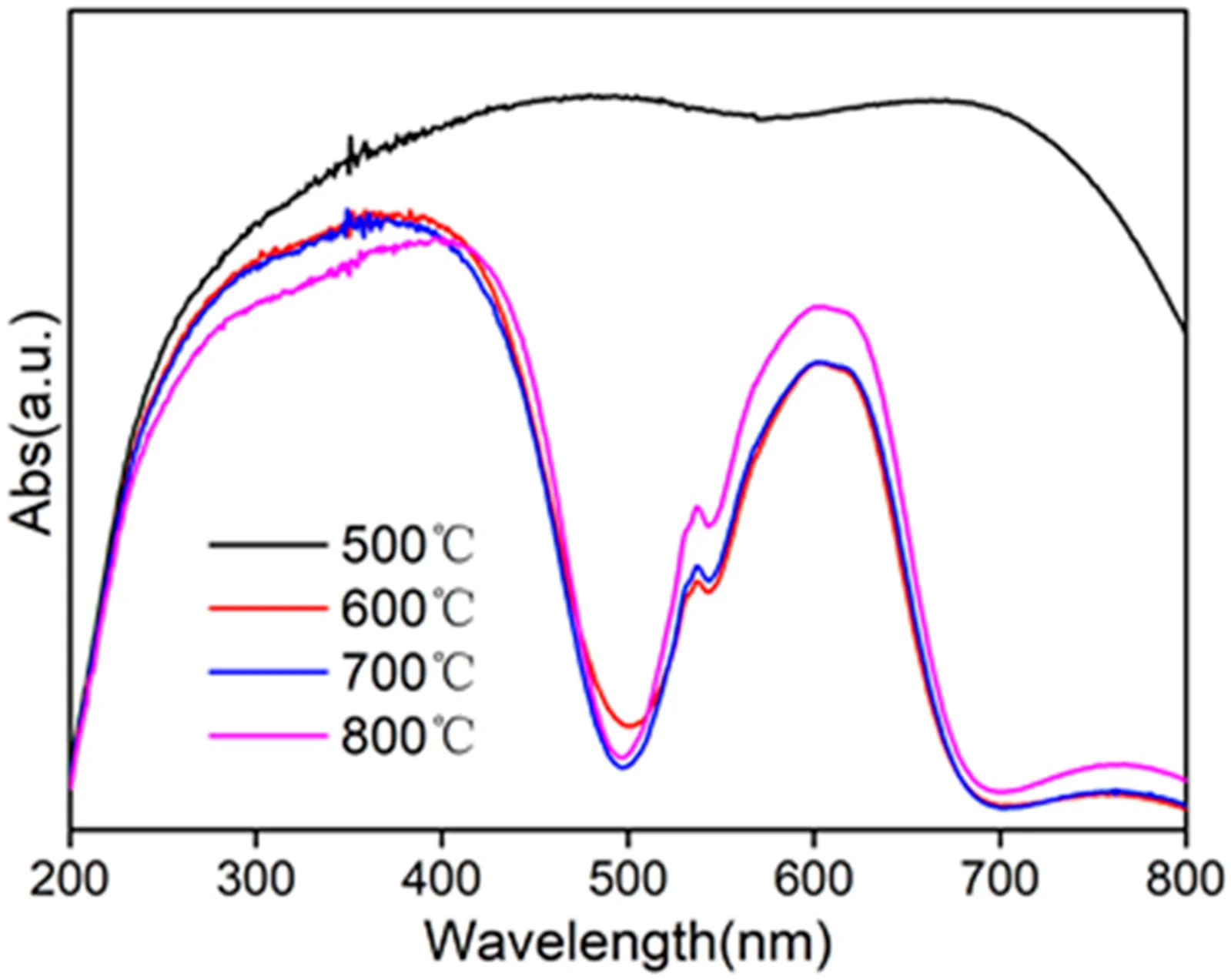
The DRS spectra of CoTiO_3_ samples with different calcination temperatures.

UV–vis diffuse reflectance spectroscopy (DRS) was conducted on CoTiO_3_ samples calcined at different temperatures (Figure 6). The sample calcined at 500 °C exhibited strong light absorption across the entire UV–vis region, which is primarily attributed to the formation of cobalt oxides during precursor calcination and the carbonization of organic matter. In contrast, when the calcination temperature was increased to 600 °C, 700 °C, and 800 °C, the resulting samples displayed markedly different light absorption characteristics in the UV–vis region compared to CoTiO_3_-500 °C. Specifically, a distinct downward trend in absorbance is observed in the range of 450–500 nm, which arises from charge transfer from O^2−^ to Ti^4+^. In addition, strong characteristic peaks are observed at 536 nm and 604 nm, ascribed to the Co^2+^ → Ti^4+^ transition resulting from the splitting of the 3d^8^ energy level of Co^2+^ ions [25].

**Figure 7:**
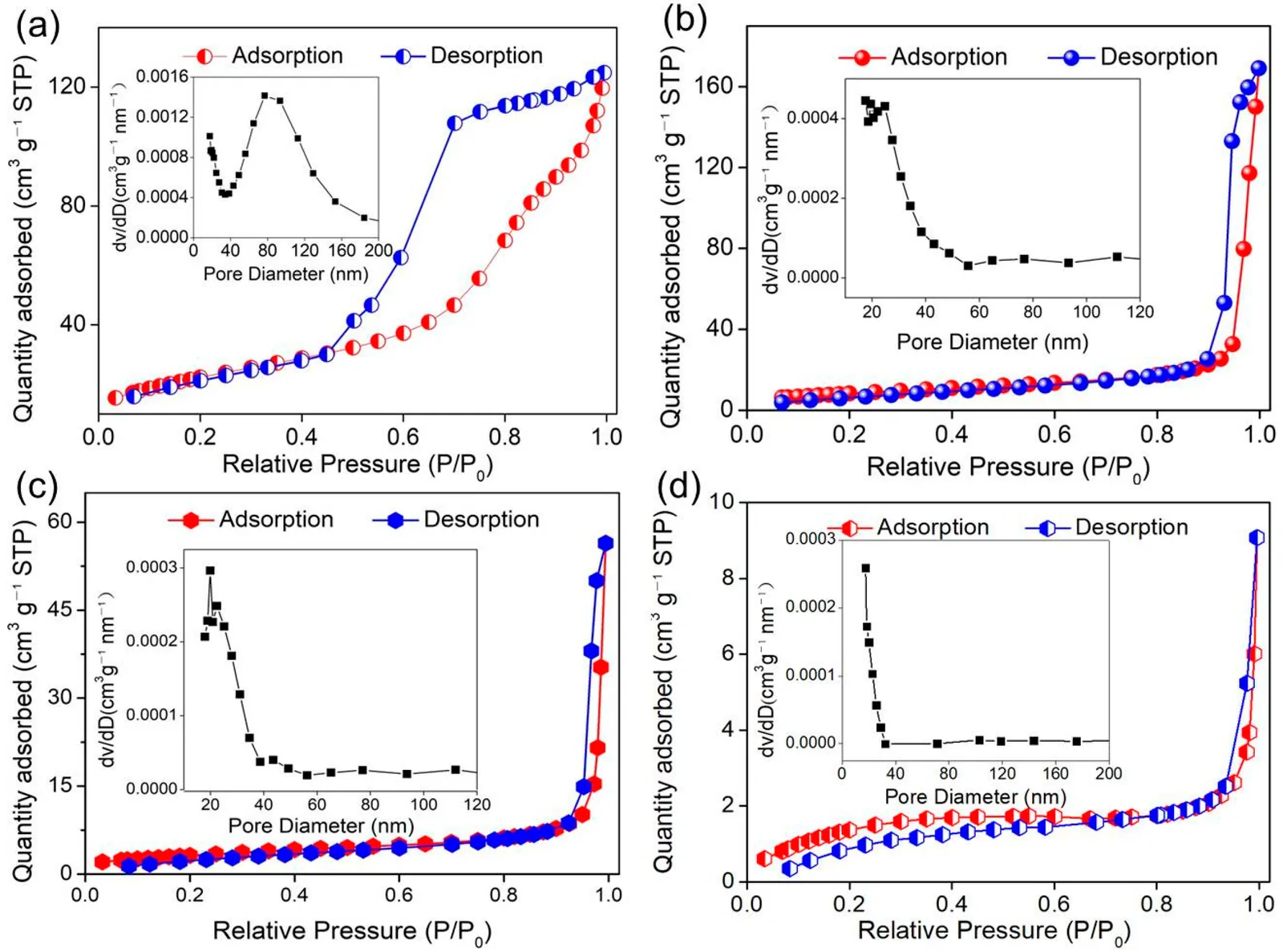
N_2_-adsorption desorption isotherms of CoTiO_3_ in different calcination temperatures, insert is the pore size distribution curve: (**a**) CoTiO_3_-500, (**b**) CoTiO_3_-600, (**c**) CoTiO_3_-700, (**d**) CoTiO_3_-800.

As shown in Figure 7, the adsorption types of the samples calcined at different temperatures all belong to Type IV isotherms. As shown in Table S1, the specific surface area follows the order: CoTiO_3_-500 > CoTiO_3_-600 > CoTiO_3_-700 > CoTiO_3_-800. Among these, CoTiO_3_-600 exhibits a relatively large specific surface area and pore volume. The CO_2_ adsorption plays a key role for CO_2_ reaction, and the CO_2_ adsorption capacity of CoTiO_3_-600 with a relatively large specific surface area was investigated. As revealed in Figure S3, the CoTiO_3_-600 shows that the maximum CO_2_ adsorption capacity of CoTiO_3_ is 5.8 cm^3^ g^−1^, which was comparable with other reported materials [26].

**Figure 8:**
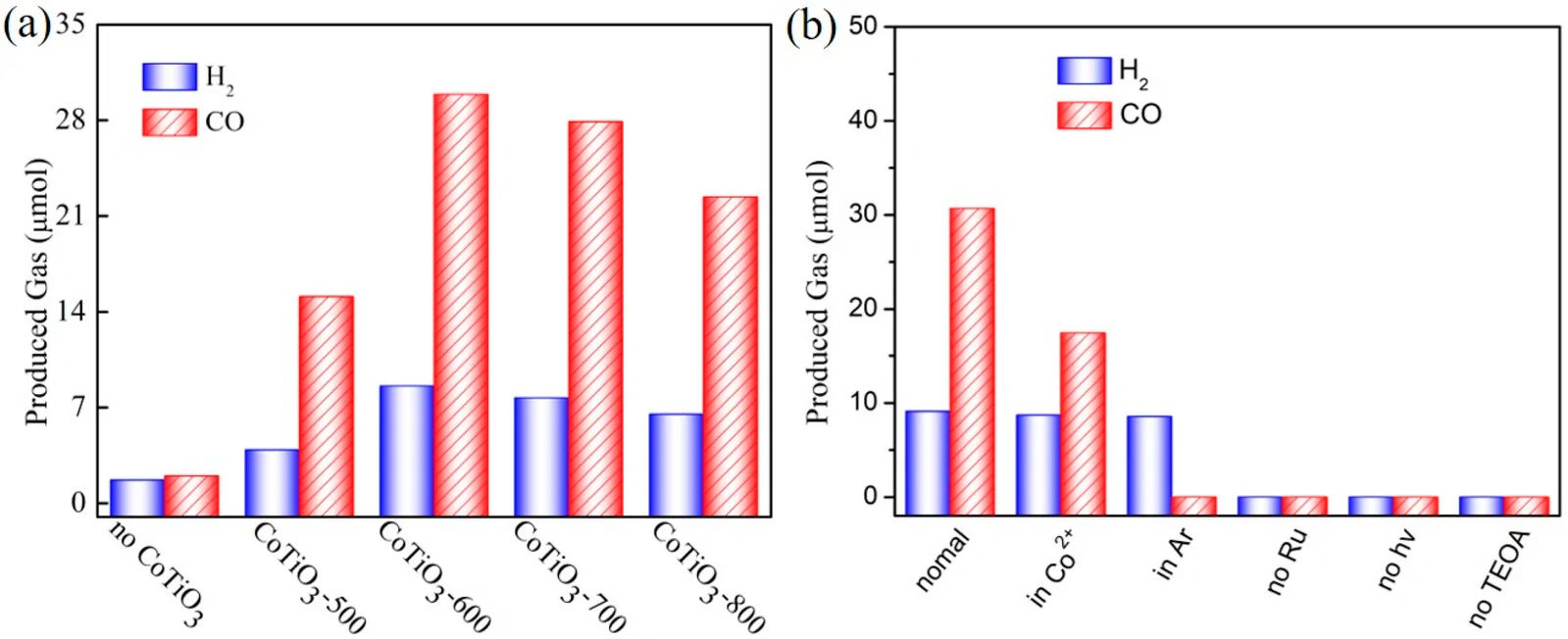
The comparison of CO_2_ activity reduction with CoTiO_3_ nanorods at different calcination temperatures (**a**). Photocatalytic reduction of CO_2_ system by CoTiO_3_ under different conditions (**b**).

Figure 8a presents the photocatalytic CO_2_ reduction performance of CoTiO_3_ samples calcined at different temperatures. In the absence of CoTiO_3_, only 2.0 μmol CO and 1.7 μmol H_2_ were produced. Upon introducing the CoTiO_3_ sample calcined at 500 °C, the yields of CO and H_2_ increased to 15.1 μmol and 3.9 μmol, respectively. When the calcination temperature was raised to 600 °C, the sample exhibited optimal photocatalytic performance, yielding 29.9 μmol CO and 8.6 μmol H_2_. However, as the temperature further increased to 700 °C and 800 °C, a decline in CO and H_2_ production was observed, which may be attributed to the decreased specific surface area and the increased average crystallite sizes of the CoTiO_3_ samples calcined at higher temperatures. Therefore, the CoTiO_3_ sample calcined at 600 °C was selected for a more detailed study of the photocatalytic CO_2_ reduction reaction system.

To investigate the relationship between reaction system parameters and catalytic performance, a series of control experiments were designed. As shown in Figure 8b, under reaction conditions without a photosensitizer, light source, or sacrificial agent, almost no gaseous products (CO and H_2_) were detected after 1 h of reaction, indicating that all three components are essential for photocatalytic CO_2_ reduction. Under visible-light irradiation, the photosensitizer is photoexcited to generate photogenerated electrons. The presence of a sacrificial agent quenches the oxidized state of the photosensitizer, thereby prolonging the lifetime of the photogenerated electrons and consequently improving the reaction efficiency. Only 8.7 μmol H_2_ was produced under an Ar atmosphere, demonstrating that CO originated from CO_2_. When Co^2+^ was added to the reaction system as a substitute for the CoTiO_3_ catalyst, 8.7 μmol H_2_ and 17.4 μmol CO were generated. To verify the stability of the catalyst, the reaction filtrate of the system containing the CoTiO_3_ sample was analyzed by ICP-MS. The concentration of Co^2+^ was found to be below 10 ppm, demonstrating the stability of the CoTiO_3_ material and its ability to significantly enhance the photocatalytic CO_2_ reduction activity. To identify the carbon source of the produced CO, isotopic labeling experiments were conducted using ^13^CO_2_ as the feedstock under otherwise identical reaction conditions. After 1 h visible-light irradiation, the evolved gas was analyzed by GC-MS (Figure S4). The results demonstrate that only ^13^CO (*m*/*z* = 29) was detected when ^13^CO_2_ served as the feedstock. This result verifies that the generated CO stems from the reductive deoxygenation of CO_2_, not from any other organic components in the system.

**Figure 9:**
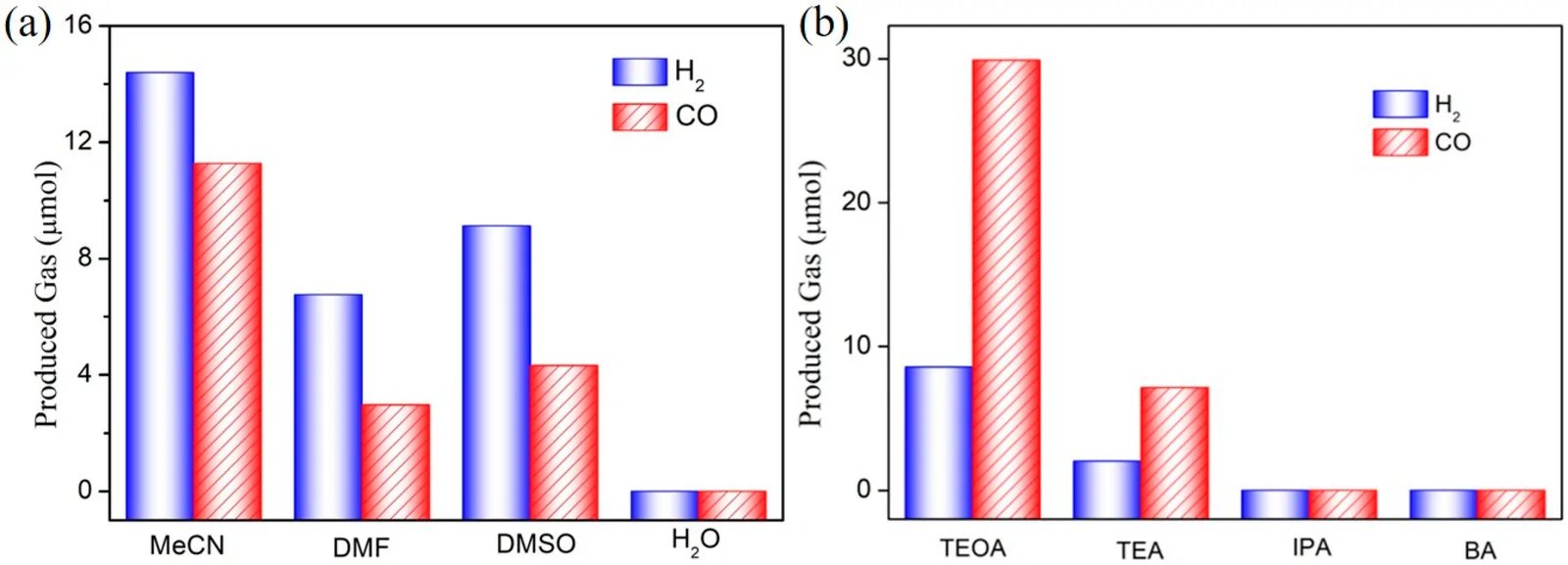
The activity of CO_2_ reduction in different solvents (**a**). The different sacrificial agents on reaction performance (**b**).

In the photocatalytic CO_2_ reduction reaction system, the reaction medium has a significant influence on the catalytic performance of the catalyst. Four kinds of aprotic solvents containing N or O atoms, MeCN, DMF, and DMSO were selected as the reaction media, and all the solvents exhibited CO_2_ reduction ability with different activity. The possible reason is that the different solvents possess different CO_2_ solubility, consequently affecting the CO_2_ reduction efficiency. CO_2_ exhibits Lewis acid–base interactions with heteroatoms such as N and O in aprotic solvents, which facilitates CO_2_ dissolution, thereby promoting its reduction. In contrast, in the protic solvent H_2_O, no gaseous products were detected (Figure 9a). which is caused by the weak chemical interactions (van der Waals forces) exist between H_2_O and CO_2_, making CO_2_ less soluble in the medium [27,28]. Among the solvents tested, MeCN gave the best activity, with a CO production rate of 11.3 μmol h^−1^, which was higher than that of DMSO (4.3 μmol h^−1^) and DMF (2.9 μmol h^−1^).

However, during the CO_2_ reduction process, when protons participate in the multi-electron CO_2_ reduction reaction, the activation energy of the reaction can be significantly reduced. The introduction of H^+^ is beneficial for the smooth progress of the reaction [29].


CO_2_ + e^−^ → CO_2_^−^                             E_0_ = −1.9 V

CO_2_ + 2H^+^ + 2e^−^ → CO + H_2_O        E_0_ = −0.53 V

Considering the above factors, MeCN was chosen as the solvent, and different amounts of H_2_O were added to further investigate the influence of mixed solvents on CO_2_ reduction performance.

As summarized in Table S2, all reaction conditions were kept constant except for the ratio of H_2_O/MeCN. Without changing the total solvent volume, different amounts of H_2_O were added to the system. When 1 mL H_2_O was added to the system, the reaction activity rapidly increased, with the yields of H_2_ and CO reaching 9.7 μmol and 20.3 μmol, respectively. When the amount of H_2_O was increased to 2 mL, the yields of H_2_ and CO reached 8.6 μmol and 29.9 μmol, with the highest CO consumption. With the increasing amount of added H_2_O, both H_2_ and CO yields decreased, indicating that an excessive amount of H_2_O is detrimental to the CO_2_ reduction reaction, which further confirms the previous hypothesis. Notably, the production of both H_2_ and CO increased significantly upon the addition of H_2_O to the system, indicating that the introduction of H_2_O enhances the catalytic activity for CO_2_ reduction.

In the photocatalytic CO_2_ reduction reaction, the type and amount of sacrificial agent may have a significant impact on the CO_2_ reduction reaction. As shown in Figure 9b, different sacrificial agents, triethanolamine (TEOA), triethylamine (TEA), isopropanol (IPA), and benzylamine (BA), resulted in different reaction activities. When TEOA was employed as the sacrificial agent, which showed the highest CO_2_ reduction catalytic activity, with gas product yields of 8.6 μmol H_2_ and 29.9 μmol CO. In contrast, no gas products were detected when IPA or BA was used.

**Figure 10:**
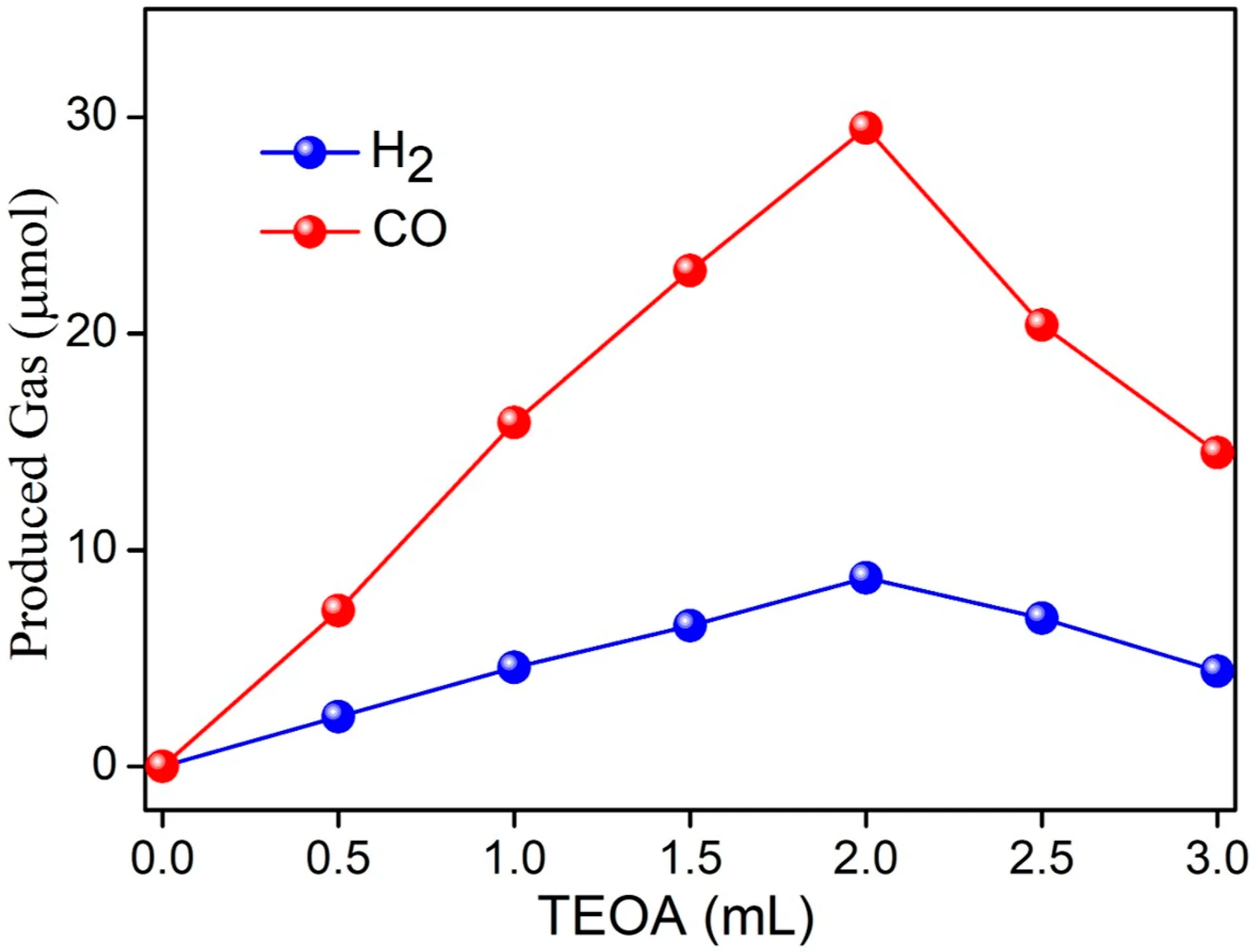
Effect of the amount of sacrificial TEOA added on the reaction performance.

As shown in Figure 10, the amount of triethanolamine (TEOA) added has a significant influence on the activity of the reaction system in the same H_2_O/MeCN solvent ratio. No products were produced without TEOA. The addition of 0.5 mL of TEOA resulted in gas yields of 2.3 μmol for H_2_ and 7.2 μmol for CO. A maximum was achieved when the amount of TEOA was increased to 2 mL, with the yields of H_2_ and CO reaching 8.6 μmol and 29.9 μmol, respectively. Upon further addition of TEOA, the yields of H_2_ and CO decreased rapidly, this may be ascribed to the excessively strong alkalinity of the solution, which affects the reduction potential of the system and is unfavorable for the CO_2_ reduction reaction. The CO evolution was comparable with other reports (Table S3).

**Figure 11:**
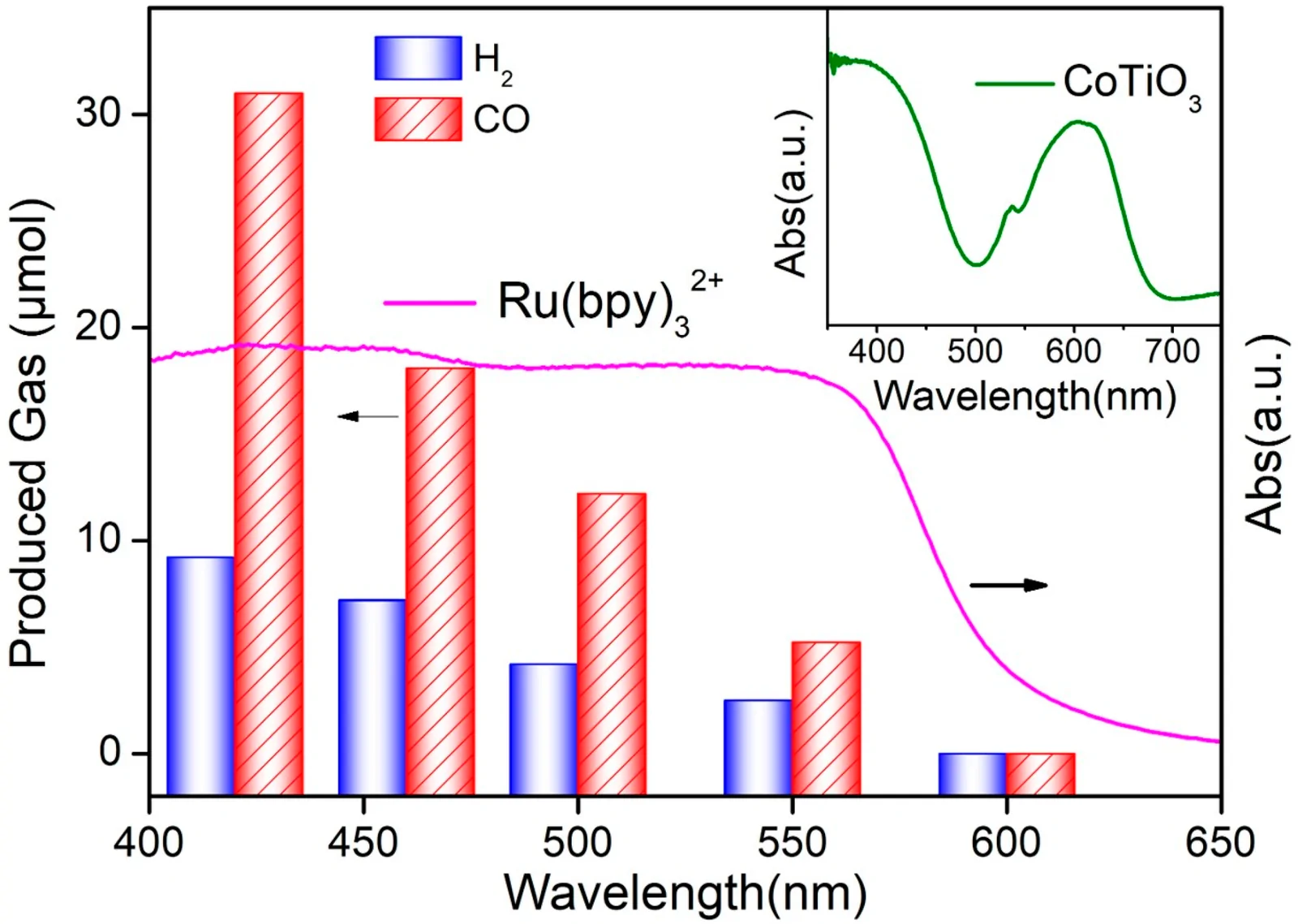
Wavelength dependence of the evolution of CO/H_2_.

Figure 11 presents the activity of CoTiO_3_ in the photocatalytic CO_2_ reduction system as a function of incident light wavelength. When different cutoff filters were used to select specific wavelength bands, the highest yields of H_2_ (9.1 μmol) and CO (31.2 μmol) were achieved with a 420 nm cutoff filter. As the incident wavelength increased, the yields of both H_2_ and CO exhibited a clear downward trend. When the wavelength was further increased to 600 nm using a 600 nm cutoff filter, no gaseous products were detected. The dependence of the generation of CO/H_2_ corresponds well to the absorption spectrum of the Ru complex, rather than that of the CoTiO_3_ material, The results revealed that the CO_2_ photoreduction catalysis was actually initiated by the photoexcitation of the Ru light harvester, forming light-stimulated electrons for successive tandem photoredox reactions.

**Figure 12:**
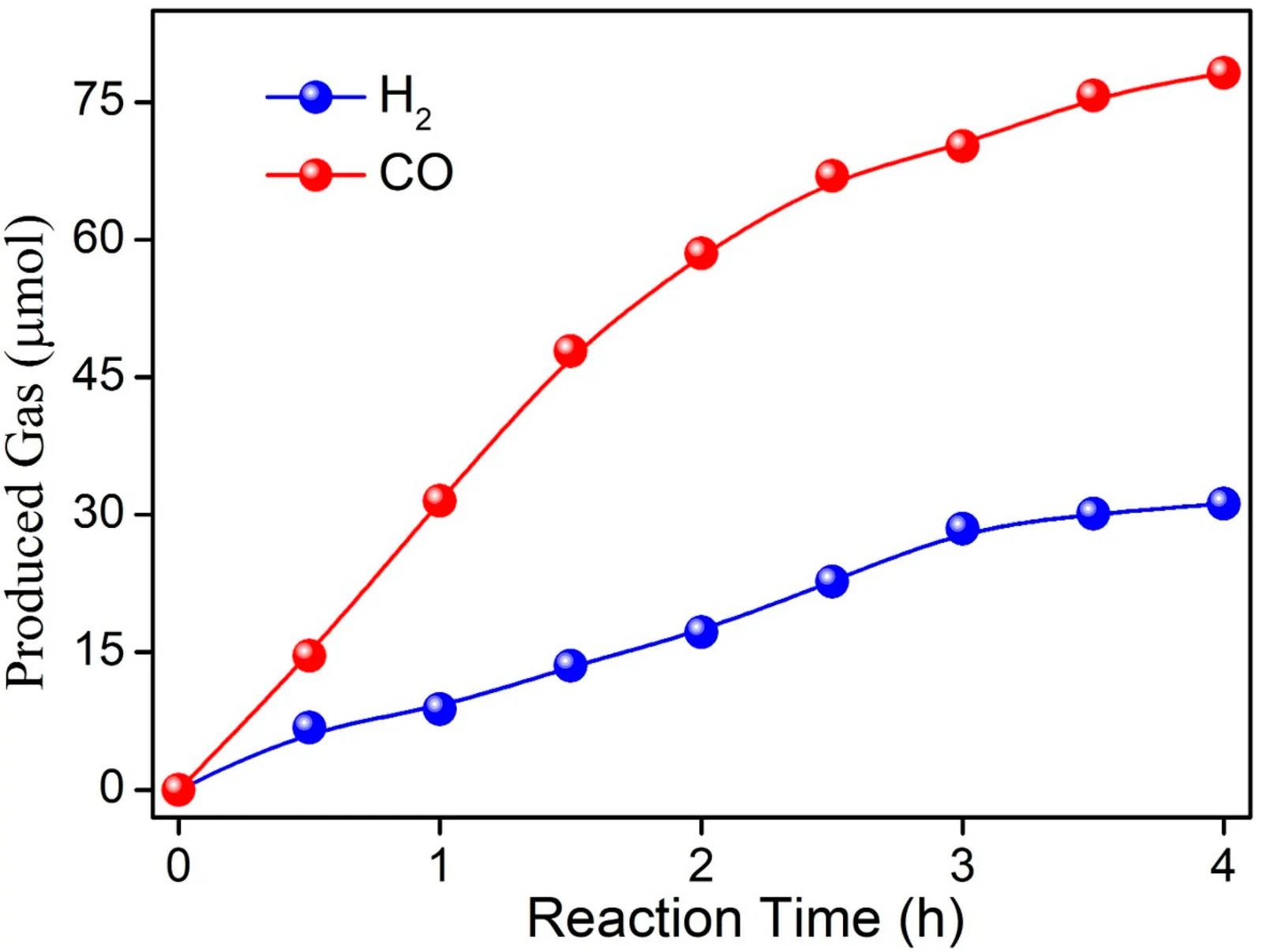
Time–production curve of photoreduction of CO_2_ with CoTiO_3_ catalyst.

Figure 12 presents the time-dependent evolution curves of CO and H_2_ production in the CoTiO_3_-catalyzed CO_2_ reduction system. During the first hour, the yields of H_2_ and CO increased almost linearly. After visible-light photocatalysis for 4 h, the H_2_ and CO evolution reached 31.2 μmol and 78.2 μmol, respectively. As is revealed, the reaction rate first increased but then reduced gradually, possibly due to the gradual deactivation of the photosensitizer Ru(bpy)_3_^2+^ during the reaction process, rather than the instability of the CoTiO_3_ cocatalyst, which will be solidly confirmed later on.

**Figure 13:**
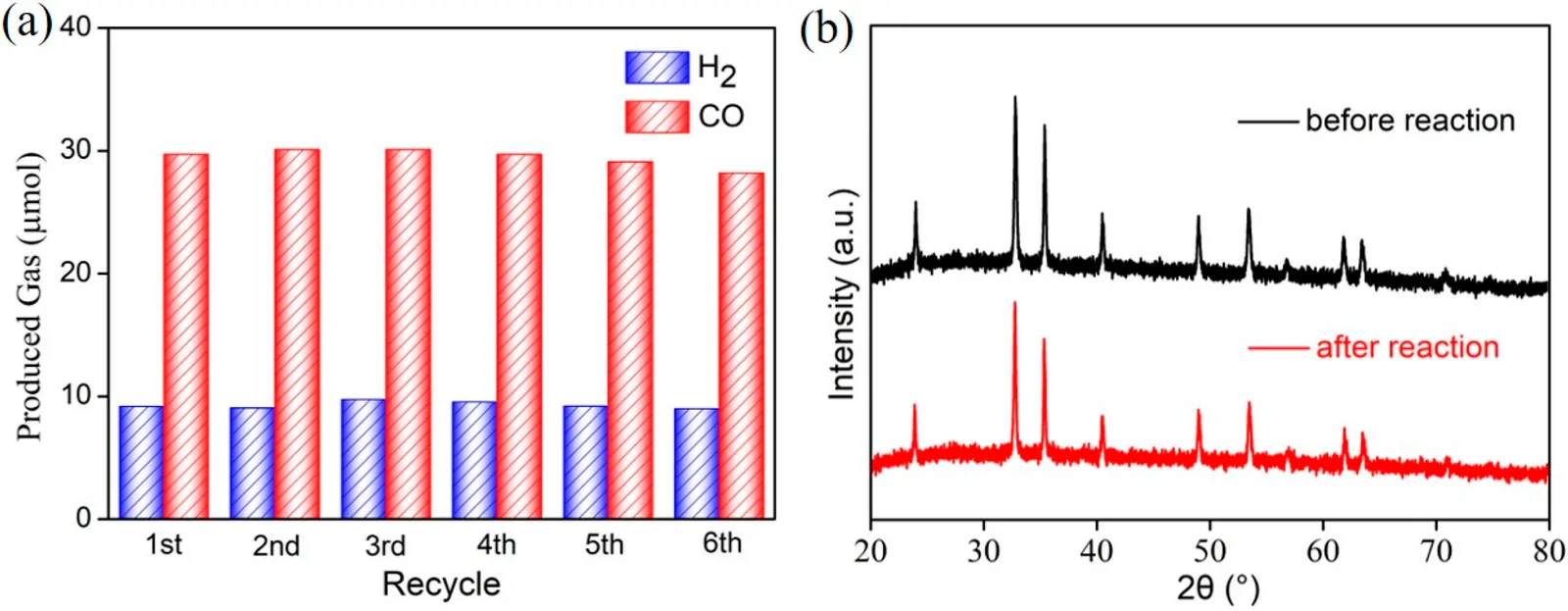
Activity stability test of CoTiO_3_ (**a**). XRD patterns of the CoTiO_3_ samples before and after reactions (**b**).

Finally, the stability and reusability of the CoTiO_3_ cocatalyst were examined, as these properties are of great importance for its further development. The results (Figure 13a) demonstrated that the CoTiO_3_ solid exhibited excellent activity stability, revealing its high reusability for the CO_2_ photofixation reaction. After the photocatalytic experiments, CoTiO_3_ was separated from the reaction mixture, washed with ethanol, and characterized by XRD. As shown in Figure 13b, no noticeable changes in the crystal structure or surface chemistry of the used sample were detected compared to the fresh CoTiO_3_ material, firmly confirming the high stability of the CoTiO_3_ cocatalyst in the present photochemical system.

**Figure 14:**
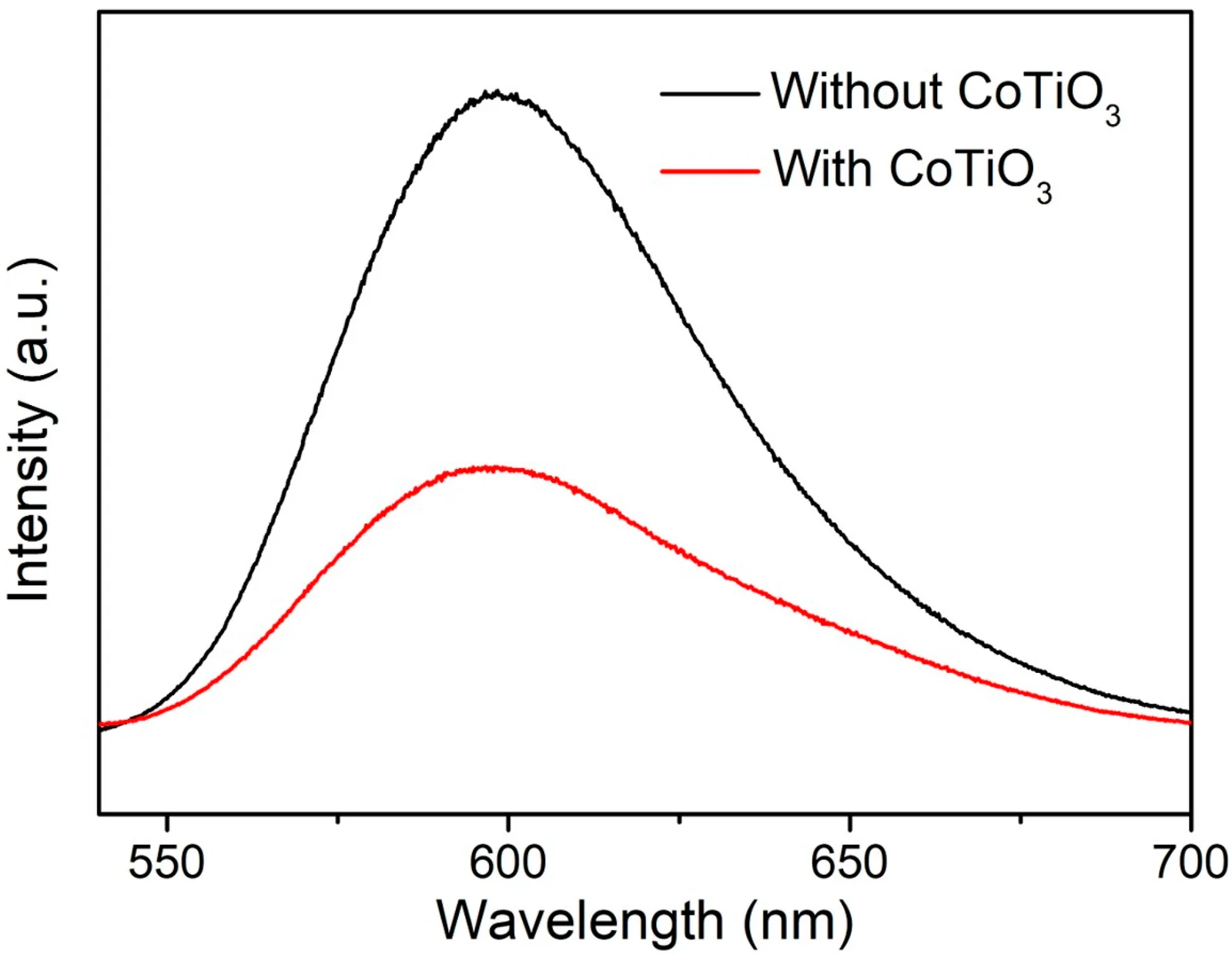
PL spectra of photocatalytic reduction with and without CoTiO_3_ sample.

To demonstrate the ability of CoTiO_3_ to facilitate rapid electron transfer in the system, fluorescence spectroscopy was performed on the reaction system. As shown in Figure 14, Ru(bpy)_3_^2+^ exhibited a broad luminescence band centered at 595 nm, which can be attributed to the transition from the surface trap states to the ground state of Ru(bpy)_3_^2+^. In contrast, the addition of CoTiO_3_ nanorods to the reaction system significantly decreased the fluorescence intensity. This observation indicates that the introduction of CoTiO_3_ facilitates rapid electron transfer in the reaction system, accelerates the CO_2_ reduction rate, and improves the efficiency of photocatalytic CO_2_ reduction.

The flat band potential of CoTiO_3_ was further examined to elucidate the reaction pathway of CO_2_ photoconversion catalysis. Mott–Schottky analysis (Figure S5) revealed a flat band potential of -0.65 V (vs. NHE, pH 7.0), indicating that CoTiO_3_ possesses a suitable redox potential to accept photoinduced electrons from the Ru dye for CO_2_ reduction to CO. In addition, H_2_ was also generated due to the direct reduction of protons by the excited electrons. Furthermore, with triethanolamine (TEOA) as the sacrificial agent, the oxidized state of the Ru complex was effectively quenched, thus completing the photocatalytic cycle.

## 3. Materials and Methods

### 3.1. Materials

Co(CH_3_COO)_2_·4H_2_O and Ti(OC_4_H_9_)_4_ (Shanghai Aladdin Biochemical Technology Co., Ltd., Shanghai, China), Ethylene glycol (Sinopharm Chemical Reagent Co., Ltd., Shanghai, China), the figures were formed by Chem 3D v22 and Origin 2025b.

### 3.2. Synthesis of CoTiO_3_

Co(CH_3_COO)_2_·4H_2_O (0.01 mol) was dissolved in 60 mL of ethylene glycol, then tetrabutyl titanate (Ti(OC_4_H_9_)_4_) (0.01 mol) was added dropwise into the above solution. The mixture was stirred until homogeneous to form a mixed solution, which was then continuously stirred for 24 h at room temperature. The resulting CoTiO_3_ precursor was washed three times with ethanol and dried in a vacuum oven at 60 °C. To obtain the crystalline CoTiO_3_ sample, the precursor was annealed at between 500 and 800 °C, which were denoted as CoTiO_3_-500, CoTiO_3_-600, CoTiO_3_-700, and CoTiO_3_-800.

### 3.3. Characterization

X-ray diffraction (XRD) was performed on a Bruker D8 Advanceinstrument (Billerica, MA, USA) (Cu Kα irradiation, λ = 0.154 06 nm, U = 40 kV, I = 40 mA, 2θ = 20–80°). The XPS spectra were acquired on a PHI Quan-tum 2000 XPS system (Chanhassen, MN, USA) equipped with a monochromatic Al Kα source and a charge neutralizer. The TEM images were captured using a JEOL model JEM 2010 EX instrument (Akishima, Japan) with an accelerating voltage of 200 kV. The BET surface area and the pore size distribution curve of the catalysts were obtained by using an ASAP 2020M apparatus (Micromeritics, Norcross, GA, USA) with N_2_ absorption at 77 K.

### 3.4. Photocatalytic Evaluation

The photocatalytic reactions were carried out in a gas-closed glass reactor containing 15 mg of photocatalyst, 8 mg of [Ru(bpy)3]2+ photosensitizer (bpy = 2′2-bipyridine), 2 mL of H_2_O, 2 mL of MeCN, and 2 mL of TEOA. After introducing high-purity CO_2_ to 1 atm, the reactor was under irradiation by using a 300 W Xe lamp (Perfectlight, Beijing, China) with a 420 nm cutoff filter. The temperature was held at 30 °C with a cooling water system, and the suspension was kept under vigorous magnetic stirring. The produced H_2_ and CO were analyzed using an Agilent 7890B GC (Santa Clara, CA, USA) equipped with a TCD and a methanizer-coupled FID. To investigate the wavelength effect, the CO_2_ reduction activity was evaluated under irradiation through different long-pass cutoff filters (420, 450, 500, and 520 nm) while keeping all other conditions unchanged. In isotopic labeling experiments, the CO_2_ was replaced by using ^13^CO_2_ as the feedstock under identical reaction conditions.

## 4. Conclusions

In summary, the efficient CoTiO_3_ nanorod cocatalyst was successfully synthesized and fully characterized. The as-obtained CoTiO_3_-600 showed superior photocatalytic activity for CO_2_ reduction to CO, achieving more than a 15-fold enhancement compared to the system without CoTiO_3_, due to its ability to facilitate rapid electron transfer, suppress charge recombination, and provide abundant active sites. Isotopic labeling with ^13^CO_2_ confirmed that the produced CO originated from CO_2_. The CoTiO_3_ cocatalyst also exhibited excellent stability and reusability, with no significant changes in structure or surface chemistry after multiple reaction cycles. This work provides an effective strategy for enhancing visible-light-driven CO_2_ photoreduction based on a cost-effective and robust ternary metal oxide cocatalyst, which will be conducive to advancing sustainable solar fuel production in the future.

## Data Availability

The original contributions presented in this study are included in the article/Supplementary Materials. Further inquiries can be directed to the corresponding authors.

## Supplementary Materials

The following supporting information can be downloaded at https://www.mdpi.com/article/10.3390/molecules31183281/s1. Refs. [27,28,29,30,31] are cited in the Supplementary Materials.
