# Supplementary material for: Boosted Photocatalytic CO2 Reduction to CO with Perovskite CoTiO3
Source: Molecules. 2026 Sep 16;31(18):3281. doi: 10.3390/molecules31183281 (PMC13609454; doi:10.3390/molecules31183281)
Supplement: Supplementary file 1 [file molecules-31-03281-s001.zip › molecules-4424019-supplementary.pdf]

Boosted Photocatalytic CO_2 Reduction to CO with Perovskite $CoTiO_3$

Junli Xu [1], Weilin Zheng [1], Wanpeng Sun [2,*] and Zhaoyu Wang [1,*]

[1] Fujian Provincial Engineering Research Center for Food Flexible Packaging Technology, Fujian Polytechnic Normal University, Fuqing 350300, China; xjl012794@163.com (J.X.); zhengweilin10@163.com (W.Z.)
[2] School of Chemistry and Chemical Engineering, Henan University of Technology, No. 100 Lianhua Street, Zhengzhou High-Tech Development Zone, Henan 450001, China
* Correspondence: wpsun@haut.edu.cn (W.S.); fjjssfxywzy@163.com (Z.W.)

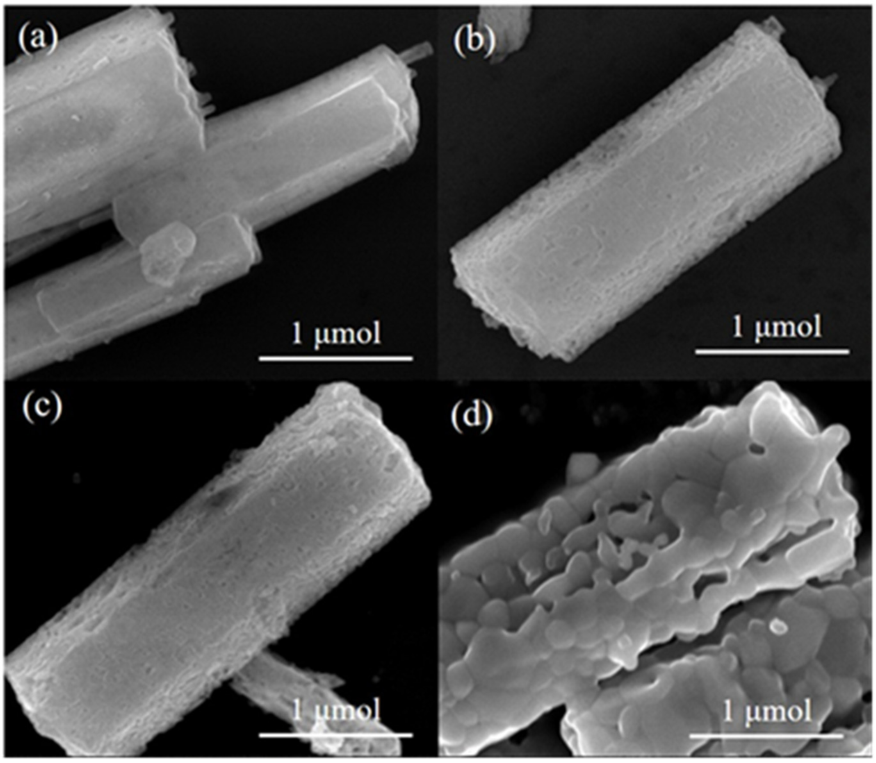

Figure S1. Typical SEM images of $CoTiO_3$ Nanorods with different calcination temperatures: (a) $CoTiO_3$-500, (b) $CoTiO_3$-600, (c) $CoTiO_3$-700, (d) $CoTiO_3$-800.

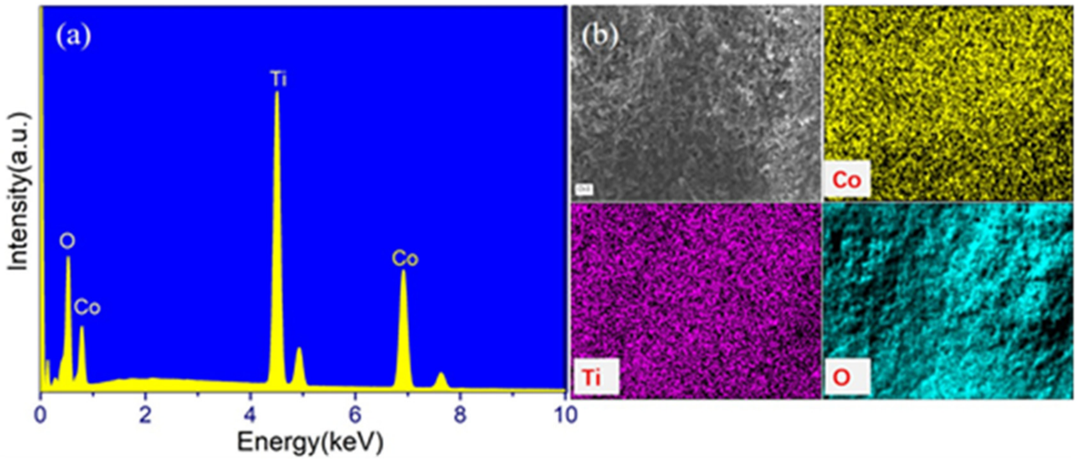

Figure S2. EDX (a) and EDX-mapping images (b) of $CoTiO_3$-600 nanorods.

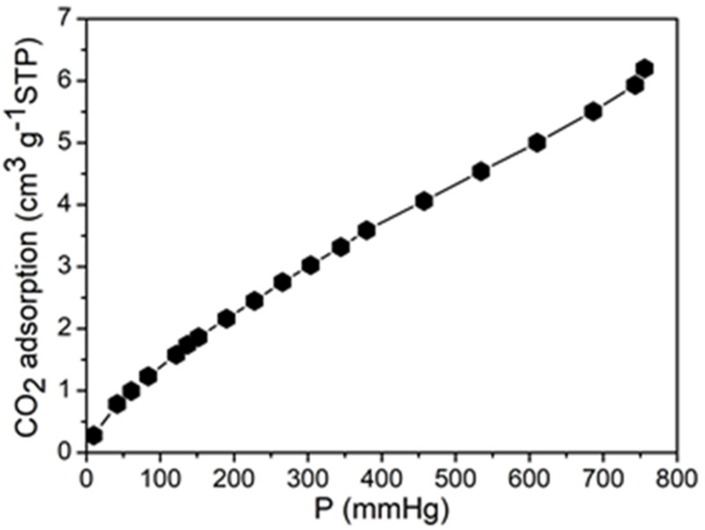

Figure S3. $CoTiO_3$ nanorods of CO_2 adsorption isotherm.

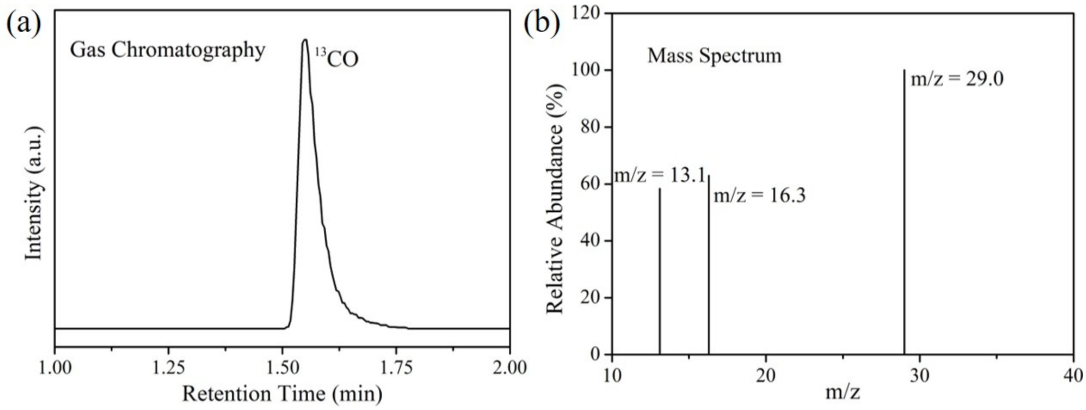

Figure S4. GC-MS analysis results of $^{13}CO_2$ tracer experiment.

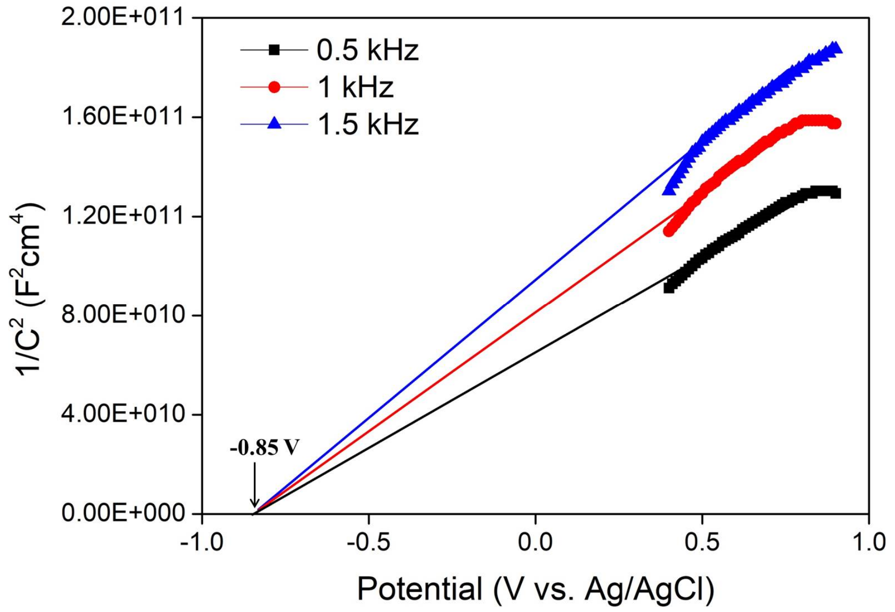

Figure S5. Mott-Schottky plot of the prepared $CoTiO_3$ material.

Table S1. The comparison of specific surface area and pore parameters of $CoTiO_3$ samples with different calcination temperature.

$CoTiO_3$ (℃)	BET (m^2/g)	pore volume (cm^3/g)	pore size (nm)
500	80.5	0.193	75
600	30.5	0.261	25
700	11.8	0.087	20
800	5.52	0.014	20

Table S2. The effect of the volumetric ratio of H_2O on the catalytic performance of the CO_2 photoreduction system.

Entry	H_2O/MeCN	H_2	CO	H_2+CO	Sel.co (%)
1	0/5	10.8	5.3	16.1	32.7
2	1/4	9.7	20.3	30.0	67.6
3	2/3	8.6	29.9	38.5	77.7
4	3/2	4.2	11.4	15.6	73.1
5	4/1	1.1	2.5	3.6	69.4
6	5/0	0	0	0	0

Table S3. the CO evolution in reported literatures

Ref	catalysts	CO evolution (μmol/g/h)
1	$NiCo_2O_4$	1458
2	$ZnIn_2S_4$-In_2O_3	307
3	In2S_3-$CdIn_2S_4$	825
4	$CuCo_2O_4$	1190
5	$MnCo_2O_4$	1377
This work	$CoTiO_3$	1303

References

1 Wang, Z. Y.; Jiang, M.; Qin, J. N.; Zhou, H.; Ding, Z. X.; Reinforced photocatalytic reduction of CO_2 to CO by a ternary metal oxide $NiCo_2O_4$. *Phys. Chem. Chem. Phys.,* **2015**, *17*, 16040-16046. DOI: 10.1039/c5cp01997d

2 Wang, S.B.; Guan, B.Y.; Lou, X.W. Construction of $ZnIn_2S_4$–In_2O_3 Hierarchical Tubular Heterostructures for Efficient CO_2 Photoreduction. *J. Am. Chem. Soc.* **2018**, *140, 15,* 5037-5040. DOI: 10.1021/jacs.8b02200.

3 Wang, S.B.; Guan, B.Y.; Lu, Y.; Lou, X.W. Formation of Hierarchical In_2S_3-$CdIn_2S_4$ Heterostructured Nanotubes for Efficient and Stable Visible Light CO_2 Reduction. *J. Am. Chem. Soc.* **2017**, *139, 48,* 17305-17308. DOI: 10.1021/jacs.7b10733.

4 Jiang, M.; Gao, Y. L.; Wang, Z.Y.; Ding, Z. X.; Photocatalytic CO_2 reduction promoted by a $CuCo_2O_4$ cocatalyst with homogeneous and heterogeneous light harvesters. *Appl. Catal. B.* **2016**, *198*, 180 – 188. http://dx.doi.org/10.1016/j.apcatb.2016.05.055.

5 Wang, S. B.; Hou, Y. D.; Wang, X. C.; Development of a Stable $MnCo_2O_4$ Cocatalyst for Photocatalytic CO_2 Reduction with Visible Light. *ACS Appl. Mater. Interfaces*. **2015**, *7*, 4327−4335. DOI: 10.1021/am508766s.
